# Training the Brain to Survive Stroke

**DOI:** 10.1371/journal.pone.0045108

**Published:** 2012-09-13

**Authors:** Jeff F. Dunn, Ying Wu, Zonghang Zhao, Sathya Srinivasan, Sirajedin S. Natah

**Affiliations:** 1 Department of Radiology, Faculty of Medicine, University of Calgary, Calgary, Alberta, Canada; 2 Department of Clinical Neuroscience, Hotchkiss Brain Institute, Faculty of Medicine, Calgary, Alberta, Canada; 3 Hotchkiss Brain Institute, Faculty of Medicine, University of Calgary, Calgary, Alberta, Canada; 4 Department of Physiology, Faculty of Medicine and Medical Sciences, University of Um-Alqura, Makkah, Saudi Arabia; University of Cambridge, United Kingdom

## Abstract

**Background:**

Presently, little can be done to repair brain tissue after stroke damage. We hypothesized that the mammalian brain has an intrinsic capacity to adapt to low oxygen which would improve outcome from a reversible hypoxic/ischemic episode. Acclimation to chronic hypoxia causes increased capillarity and tissue oxygen levels which may improve the capacity to survive ischemia. Identification of these adaptations will lead to protocols which high risk groups could use to improve recovery and reduce costs.

**Methods and Findings:**

Rats were exposed to hypoxia (3 weeks living at ½ an atmosphere). After acclimation, capillary density was measured morphometrically and was increased by 30% in the cortex. Novel implantable oxygen sensors showed that partial pressure of oxygen in the brain was increased by 40% in the normal cortex. Infarcts were induced in brain with 1 h reversible middle cerebral artery occlusions. After ischemia (48 h) behavioural scores were improved and T2 weighted MRI lesion volumes were reduced by 52% in acclimated groups. There was a reduction in inflammation indicated by reduced lymphocytes (by 27–33%), and ED1 positive cells (by 35–45%).

**Conclusions:**

It is possible to stimulate a natural adaptive mechanism in the brain which will reduce damage and improve outcome for a given ischemic event. Since these adaptations occur after factors such as HIF-1α have returned to baseline, protection is likely related more to morphological changes such as angiogenesis. Such pre-conditioning, perhaps with exercise or pharmaceuticals, would not necessarily reduce the incidence of stroke, but the severity of damage could be reduced by 50%.

## Introduction

Stroke is currently the third leading cause of death in western societies [1]. To reduce the impact on society, one must either reduce the number of strokes, the severity of the stroke or improve recovery from stroke. This paper relates to reduction of the severity of the stroke once it occurs. Since a stroke is an ischemic (low flow) event, the tissue is starved of nutrients including oxygen, which leads to hypoxia. Many species are hypoxia tolerant, and so it is reasonable to consider whether the broad spectrum of transcriptional changes that occur on exposure to hypoxia could induce adaptive protection against hypoxic/ischemic damage in the human brain.

When exposed to hypoxia, there is an increase in hypoxia inducible factor (HIF-1α) content and a host of “downstream” genetic events which serve to improve the capacity of the tissue to survive low oxygen conditions. Hypoxia acclimation includes increased capacity to supply oxygen, remove end-products and produce energy from anaerobic means. These events include an increase in hematocrit (erythropoiesis), vascular density (angiogenesis), glycolytic capacity and glycogen content [2], [3], [4], [5], [6], [7]. We use the term acclimation (or acclimatization) for the changes which would occur within the lifetime of an animal because in the comparative physiology literature the term adaptation refers to changes to the species over generations.

We hypothesized that this plasticity could be harnessed to improve outcome from pathological conditions such as stroke, which involve hypoxia and ischemia. Should this be validated, then it opens avenues for potential improvement of stroke outcome (independent of direct hypoxia exposure). One potential avenue for improving outcome which may be similar in mechanism would be to upregulate HIF-1α. Stabilization of HIF-1α allows HIF to dimerize and to stimulate the hypoxia response genes [7]. There is a significant literature on hypoxic/ischemic preconditioning [8], [9]. A range of mechanisms are proposed in preconditioning which could impact stroke severity including, but not limited to, a reduction of inflammation [10], [11], histamine regulation [12], endothelial reactivity [11], reactive oxygen species [13], increased vascular endothelial growth factor (VEGF) [14], [15] and increased erythropoietin [16]. Cell culture studies indicate that preconditioning can occur through intracellular genetic modifications [13]. Pharmaceutical interventions which artificially increase HIF-1α concentration are being investigated for their stroke protection characteristics [17]. Another paradigm might be specific exercise regimens which could induce HIF-1α [18].

Hypoxic/ischemic preconditioning studies are largely of short duration (up to a few hours). However hypoxia acclimation is known to require days to weeks [4], [19], [20]. Inducible factors such as HIF and VEGF have returned to baseline by three weeks post stimulation [21] and so mechanisms which require direct action of these cytokines may no longer be functioning. Longer duration acclimation would allow time for significant biochemical, physiological and morphological changes including increased capillary density. In a study using periodic intermittent hypoxia for 2 weeks, it was shown that both stroke volume and inflammatory reactions were reduced–even up to 8 weeks after cessation of the stimulus [11].

We pre-adapted (acclimated) rats to hypobaric hypoxia for 21 days and followed with a 1 h reversible ischemia. Outcomes were measured using MRI for stroke volume, histological measures for inflammation and behaviour testing. Hypoxia acclimation was assessed by measuring cortical partial pressure of oxygen (PtO_2_), and vascular density. We note that most studies of laboratory based hypoxia acclimation are undertaken at or near sea level while this study was undertaken at approximately 1000 m in Calgary. We included PtO_2_ and vascular density measurements to confirm that there was hypoxia acclimation even if the animals have been living at moderate altitude.

## Materials and Methods

### Ethics Statement

Procedures were approved by the Animal Care Committee of the University of Calgary and conformed to the guidelines established by the Canadian Council on Animal Care.

### Animals

Male Wistar rats (150–200 g, 6 weeks old from Charles River, Québec, Canada) were maintained in a 12∶12-h light-dark cycle with access to water and food *ad libitum.*

### Study Design

Male Wistar rats were used. One study examined the impact of chronic hypoxia on outcome from ischemia. The control group (n = 14) was handled similarly to the hypoxic group (n = 11) without hypoxic pre-acclimation. Hypoxic rats were kept in chambers at 330 mmHg, which is approximately ½ of the atmospheric pressure in Calgary and so the pressure is abbreviated for the paper as ½ atm. Controls were kept in the same room. Behavioural testing was done on the hypoxia group before placing them in chambers. Chambers were returned to room pressure once a week for cleaning for 30 minutes. Acclimation was done for 21 days. After 24 h under room pressure conditions (to allow respiratory patterns and cerebral blood flow regulation to return to normal) [22], behavioural testing was conducted, and the surgery undertaken for ischemic induction. There was no “sham” ischemia as we are comparing results between hypoxia and non-hypoxia adapted conditions. Behavioural testing was redone at 24 h and 48 h after ischemia. After the 48 h behaviour test, MRI was undertaken for lesion volume and animals sacrificed by perfusion fixation under anesthesia for histological analysis.

#### Animal numbers

Due to technical issues related to either surgery or MRI access, the animal numbers for different measures were variable: for behaviour (n = 15 control, n = 16 hypoxia acclimation), for immunohistochemistry (n = 14 control, n = 15 hypoxia acclimation), for MRI stroke volumes (n = 14 control, n = 11 hypoxia acclimation) and for blood gasses (n = 15 control, n = 15 hypoxia acclimation).

Parallel studies were undertaken to measure vascular density and PtO_2_ to show the influence of hypoxia acclimation on vascular density and oxygenation. This was a small study to confirm that such adaptation occurred under the conditions of this study. Five animals were implanted with PtO_2_ sensors and had PtO_2_ measured before and after acclimation. Another group had assessment of vascular density. One control animal had a failed infusion, resulting in n = 3 controls and n = 4 for hypoxia acclimation. Vascular density was not measured in the main stroke study as the key time point was the time of ischemia, but the study design did not allow for sacrifice at that time.

### Middle Cerebral Artery (MCA) Occlusion

MCA occlusion was performed as previously described [23]. While under 2% isoflurane anaesthesia (42% O_2_, 56% N_2_), the tail artery was cannulated for blood pressure measurements. The right common carotid artery was isolated and a ligature placed around it. A craniotomy (2 to 3 mm) was made over the right MCA 2–3 mm anterior to the union of the zygomatic arch and the temporal bone. The right common carotid was occluded with the ligature. The MCA was blocked using a microclip (#1, Codman). The tightening of the ligature was considered the initiation of ischemia. Anesthesia was discontinued for ischemia (60 minutes). Regional cerebral blood flow was estimated by a laser Doppler flowmeter, placed over the estimated center of the infarct. Blood pressure, blood gases, plasma glucose, and hematocrit were measured prior to, and after ischemia. A feedback-controlled infrared lamp was used to maintain core temperature (target of 37.3°C) during ischemia and until 1 hour post-ischemia.

### Behavioural Testing

Behavioural testing of unilateral forelimb disability [24], [25], [26], using two tests sensitive to damage in the sensorimotor cortex, was undertaken before infarct, as well as 1 and 2 days post-infarct. In the bilateral asymmetry test (BAT), a small, standardized piece of tape is placed on one forepaw. The animal has a natural desire to remove the tape. The time from tape placement to successful removal is recorded for each front limb. If animals were too weak to remove tape after 5 minutes, then 5 minutes was recorded. In healthy animals, there is little or no difference in the time needed to remove tape from paws from alternate sides [26]. An animal’s score is the difference (affected-unaffected forepaw) in time needed to remove the tape. The cylinder test scores exploratory movements when animals are placed in a vertically oriented cylinder for 10 minutes. Normally rats use each forelimb equally, often for bracing, while exploring the cylinder. Each forelimb contact with the cylinder wall was recorded. If both forelimbs touched the wall simultaneously, it was counted as one contact for each forelimb. The asymmetry, or ratio, score was obtained as affected forelimb/(affected + unaffected) for each rat [25]. These tests are related to cortical areas associated with damage in this model, and are independent of the extent of test experience [24], [25], [26].

### Magnetic Resonance Imaging (MRI)

A 9.4T horizontal bore MRI and a 35 or 45 mm quadrature birdcage coil were used with a T2 weighted spin echo sequence to quantify lesion volume (TR/TE = 1000/35 ms, FOV = 3×3 cm, matrix  = 256×256 pixels, slice thickness = 1 mm). MRI was undertaken approximately 48 h after onset of ischemia. Animals were anesthetized with isoflurane and core temperature was controlled using regulated air flow, and a feedback to a rectal probe. A femoral artery catheter was inserted for obtaining blood gas samples (measured with a Perkin Elmer cooximeter Stat Profile CCX, Nova Biomedical Corporation, USA). Infarct volumes were calculated by summation of the voxel volumes in multislice MRI. Using ImageJ software [27], regions of interest were drawn around the hyperintense signal (bright) voxels of the ischemic region, and the whole hemispheres, by a reader blinded to the subject type. The number of voxels in each slice containing hyperintense (infarcted) tissue were summated and converted to a volume knowing the voxel size. The hemispheric edema volume ratio was calculated as (ipsilateral volume-contralateral volume)/contralateral volume of brain [28]. Infarct volume ratio was calculated as the volume of infarct/(contralateral hemispheric volume-hemispheric edema volume) ratio [28].

### Animal Perfusion and Tissue Preparation

Animals were anaesthetized for sacrifice with intra-peritoneal ketamine/xylazine (10 mg/100 g body weight, Bimeda-MTC Animal Health Inc, Cambridge ON, Canada). Fixation was done by cardiac perfusion through the left ventricle and the right atrium was incised. First 250 ml of cold saline was infused followed by 150 ml of 10% formalin (Sigma Inc. MO, USA). The absence of color in the effluent confirmed proper perfusion. Brains were removed, paraffin embedded, and cut into 6 µm-thick coronal sections.

### Histological Staining

#### Antisera

A mouse monoclonal antibody against rat T lymphocyte (Pan-T, 1∶200, Hycult Biotechnology) was used to identify T lymphocytes. Rat microglia and macrophages were immunostained using the mouse monoclonal antibody ED1 (1∶200, Serotec). ED1 antibodies label most rat macrophages, peripheral blood monocytes, and activated microglia [29].

#### Immunostaining procedure

Sections were incubated overnight at 4°C in the first antibody in PBS 0.1 mol/L containing 2% BSA, 0.3% Triton X-100. The antigen-antibody reaction was detected by incubation with the secondary (CY3)-conjugated goat anti-mouse IgG or FITC conjugated antibodies (1∶50 to 1∶100 dilutions, Jackson Immuno-Research Laboratories, Inc., West Grove, PA, USA) for 1 h at room temperature. Sections were washed (3×10 min) with PBS, and mounted in bicarbonate-buffered glycerol (pH 8.6). Omission of the secondary antibody from the IgG staining or use of normal mouse serum in place of the primary antibody served as the staining controls. Stained brain sections were then visualized with an Olympus fluorescence microscope (BX61).

### Image Analysis and Quantification of Inflammatory Cells

A digital camera (Microfire A/R, Optronics, USA) interfaced for Windows digital imaging system (StereoInvestigator 7.50.4, MicroBright Field Inc., Williston, Vermont, USA) was used to convert the microscopic images of different brain regions into digitized images. To measure the density of ED1-positive cells and pan-T cell lymphocytes in the brain, five sections from each of the stroke lesion edges or stroke core regions in each animal were imaged using a 10x and 20x objective lens using an Olympus BX61 fluorescent microscope.

The numbers of cells in 20x images were counted manually, using the StereoInvestigator program (Microbrightfield Vermont). All images were analyzed using the same calibration. All data were analyzed blinded to the sample type. Means were calculated for each animal individually before the means were determined for the group. The results are expressed as the mean number of cells per field (0.264 mm^2^).

### Tissue Partial Pressure of Oxygen (PtO_2_) Measurements

An implantable fiber optic with a fluorescent material that is sensitive to PtO_2_ was used to measure cortical PtO_2_ using a method developed in our laboratory [30]. The method uses a fluorescent compound embedded in material placed at the tip of a fiber optic lead. The leads are connected to the Oxylite fluorescent PO_2_ sensor (Oxford Optronics, UK). The implanted fiber was approximately 250 µm in diameter and was implanted 1.8 mm below the surface of the bone, into the cortex under isoflurane anesthesia. The implant was encased in a plastic holder which was fixed to the skull with screws and dental cement. The implantation and surgery [30] were done at least a week before data were recorded, in order to give time for the tissue to heal around the implant. The implant had a protruding fiber which could be connected, through a fiber optic lead, to the Oxylite system when recording was to be undertaken. Data were recorded at 2 Hz for 5 minutes to obtain an average value. Cortical PtO_2_ data were recorded from awake, unrestrained rats breathing room air. After attaching the recording lead, animals were placed in a container and left undisturbed for at least 15 minutes before obtaining data on PtO_2_.

### Tissue Vascular Density Measurements

This method is based on a previously published technique [2]. Fluorescein isothiocyanate dextran (0.1 ml of 50 mg/ml, w/v in PBS; FITC, Sigma-Aldrich, St. Louis, MO) was perfused transcardially and circulated for 10 minutes while under isoflurane anesthesia. Brains were immersion fixed in 4% paraformaldehyde overnight at 4°C in the dark, washed with PBS and cryoprotected in 30% sucrose for 48 h. Brains were embedded in TissueTek OCT (Sakura Finetek USA, Torrance, CA, USA) and frozen in pre-cooled 2-methylbutane. 50 µm cryosections were obtained and random sections selected at every fifth section within the region of interest. Confocal image stacks were obtained within the region of interest at 2 µm step size using an Olympus FV300 microscope. Stacks were analysed to measure capillary density based on the principles described by Mouton et al., 2002 [31] using the Space Balls probe in the StereoInvestigator software (Microbrightfield Inc, Williston, Vermont, USA). Length per unit volume (Lv) and length per unit area (L) were calculated using the space balls probe. Sections were analysed in a blinded fashion.

### Statistical Analysis

Statistical analysis for behaviour was done using a two way repeated ANOVA with a Tukey-B post-hoc test for multiple comparisons. For behavioural studies, the 24 h and 48 h data were compared with the pre-ischemic data**.** One way ANOVA was used for analysis of volume of stroke lesions, density of ED1 positive cells and pan-T cell lymphocytes (control vs. hypoxia acclimated). A paired t-test was used to compare PtO_2_ values pre- to post acclimation and an unpaired t-test was used for comparing vessel density in the non-acclimated control vs. hypoxia acclimated group. A *p* value of less than 0.05 was considered to be significant.

## Results

**Figure 1:**
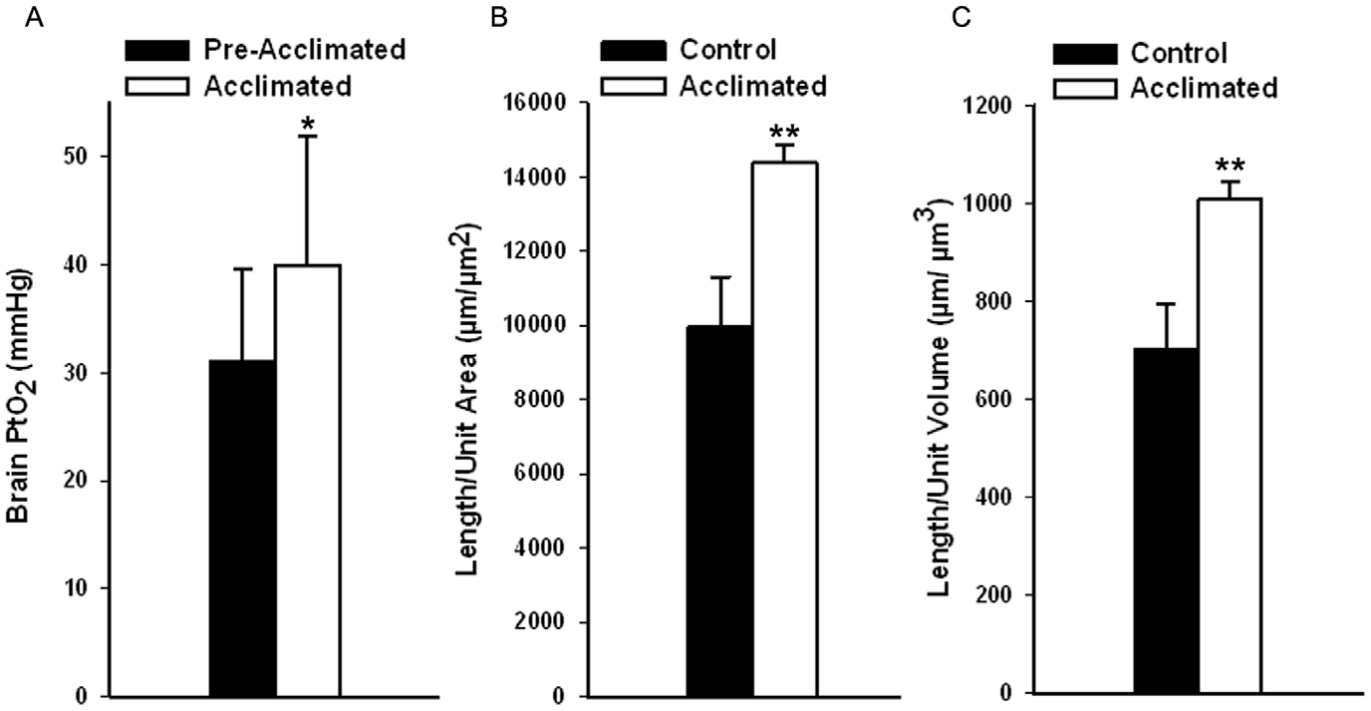
Partial pressure of oxygen in tissue (PtO_2_) measurements in brain parenchyma and capillary density data showing increased oxygen delivery after acclimation. A. Brain (cortex) PtO_2_ measurements from the same animals both pre- and post-acclimation (n = 5, mean±S.D. paired t-test comparing pre- and post-acclimation, p<0.05). **B.** Capillary length per area of cortex before and after acclimation **C.** Capillary length per volume of cortex before and after acclimation. Measurements are from the dorsolateral cortex in the CA4-CA5 region and show the increased capillary density associated with acclimation to hypoxia. Control, non-acclimated subjects are compared with acclimated subjects (mean±S.D., *p<0.05, **p<0.005).

Hypoxia acclimation resulted in increased oxygen carrying capacity. The acclimated values were significantly higher than in the non-acclimated group (p<0.05) for total hemoglobin (174±22 g/l vs. 124±11, mean±S.D., n = 15) and for hematocrit (52±7% vs. 37±3%, mean±S.D., n = 15). Capillary density and tissue oxygen levels were also increased. Capillary density (based on capillary length per volume and length per area of cortex), showed a significant increase (p<0.05) of 30% in the cortex with acclimation (**Fig. 1**). The PtO_2_ in the cortex was measured in the same animals both pre- and post-acclimation, and also showed a significant increase (p<0.01) of 40% (**Fig. 1**).

**Figure 2:**
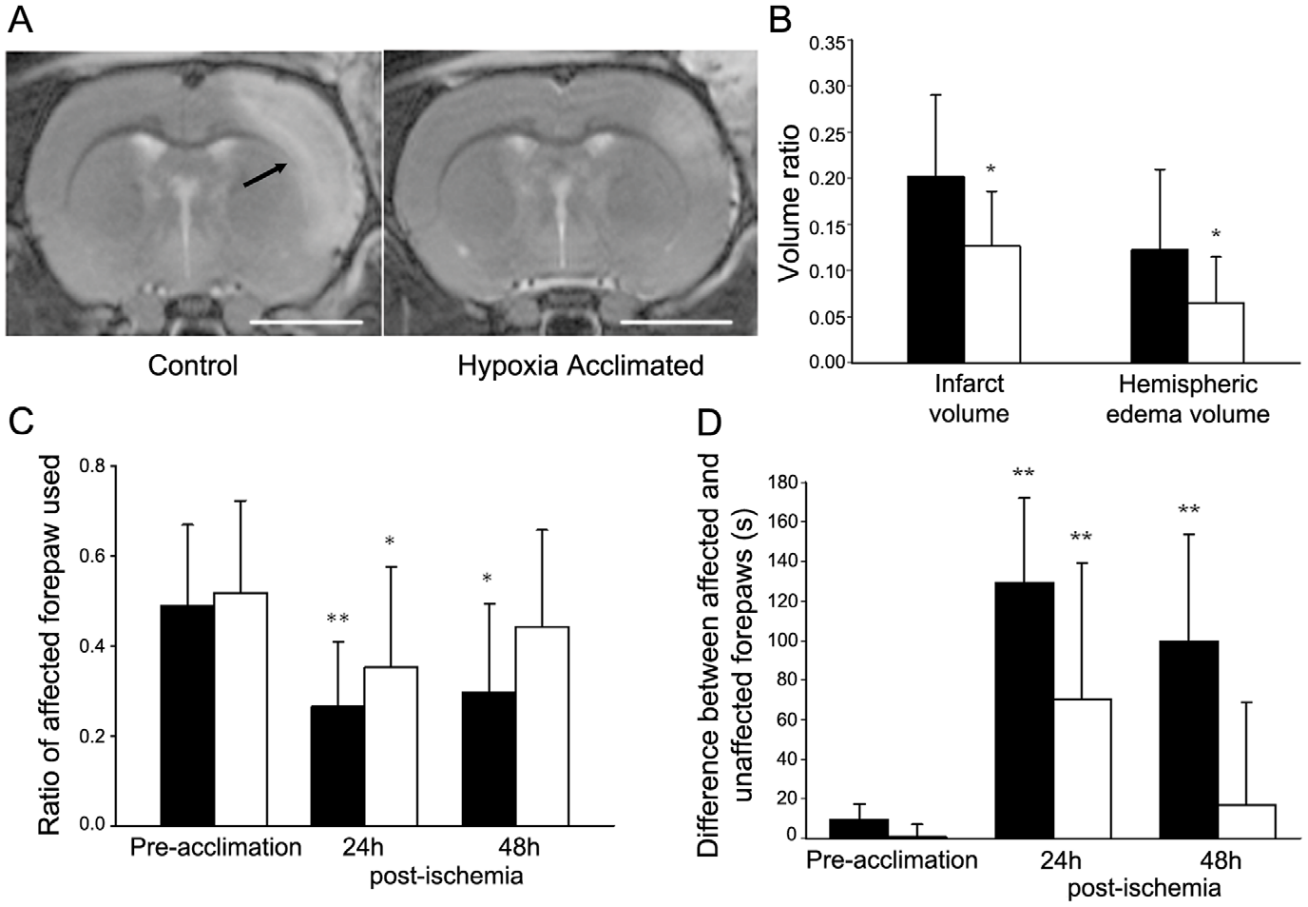
Stroke protection resulting from hypoxia acclimation. **A.** Representative T2 weighted MRI’s at 48 h post-ischemia in hypoxia acclimated, and control rats. The bright (hyperintense) area is indicative of damage or edema (scale = 5 mm). **B.** Indices of reduction in stroke volume measured using T2w MRI. Both infarct volume and edema volume ratio were significantly reduced (see methods for calculation) (mean±S.D, *p<0.05). **C.** Behaviour testing with the cylinder test. Control (closed bars) and acclimation group (open bars). **D.** Behaviour testing with bilateral asymmetry tape test. Pre-ischemic behaviour was done 24 h before ischemia. Acclimation alone did not change behaviour scores. Statistical comparison was done comparing results post-ischemia with pre-ischemic results. Acclimated and non-acclimated groups showed impairment with both tests 24 h after infarct. By 48 h, the acclimated group showed no significant difference from pre-ischemic values with both tests. Controls still had significant behaviour deficits at 48 h. (mean±S.D. *p<0.05, **p<0.001) compared to pre-ischemia.

Chronic hypoxia acclimation provided significant protection against ischemic damage. T2 weighted MR images obtained 48 h post-ischemia showed the infarction as a hyperintense region. Pre-acclimated animals showed a smaller lesion (**Fig. 2A**). The absolute lesion volume was reduced by 52% in the hypoxia acclimated rats (66.4±45 mm^3^, mean±S.D.) vs. controls (138.9±85 mm^3^, mean±S.D.). This reduction was significant (p<0.05) whether using infarct volume ratio or hemispheric edema volume ratio [28] (**Fig. 2**).

Bilateral asymmetry and cylinder score tests were used to monitor sensorimotor function before the stroke, as well as at 1 and 2 days post-ischemia. With unilateral cortical damage, the time needed to remove the tape increases unilaterally [26]. The cylinder test scores the number of times the animal explores the cylinder wall with each forelimb and the result is calculated as the ratio of touches of the affected forelimb over the total number of touches. The animal selectively uses an unimpaired forelimb for exploring and weight bearing. There was no significant difference between pre-acclimated and post-acclimated behaviour scores before ischemia. Hypoxia acclimated rats had no significant behavioural impairment by 48 h post-ischemia, while the control group was still showing significant impairment (**Fig. 2** **C,D**).

The protective effect included a reduction in inflammation. Immunohistochemistry was done on samples obtained at 48 h post-ischemia for rat T-lymphocytes (Pan-T), and microglia/macrophages (ED1) [29]. Some Pan-T positive staining indicated that lymphocytes were present ipsilaterally in the undamaged parenchymal cortex. No ED1 positive staining macrophages were seen outside the ischemic region.

**Figure 3:**
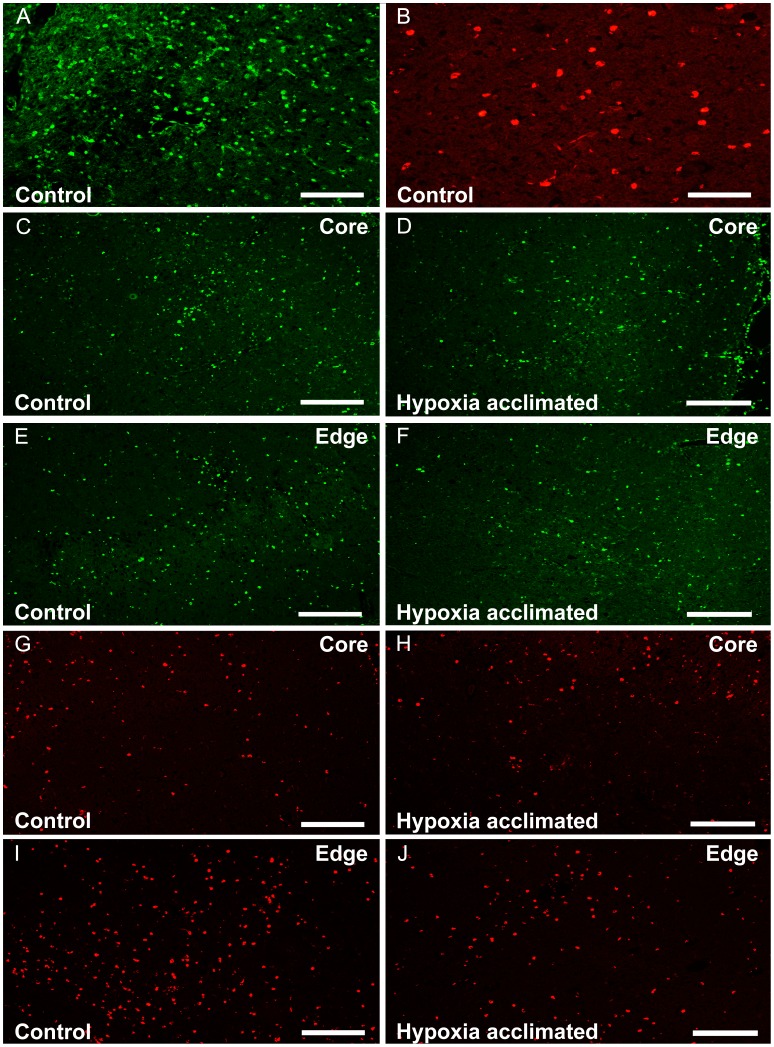
Immunostaining of infiltrating cells within the infarct of non-acclimated (control) and hypoxia acclimated animals. A,B Example control images obtained with a 20x objective, the magnification used for cell counting (scale  = 100 µm) **A** Rat Pan-T cell immunostaining for T-cell lymphocytes. **B** Rat ED1 staining for microglia/macrophages (**C–D**) 10x images of Pan-T cell immunostaining demonstrating the progression of T-cell lymphocytes from the periphery to the core of the infarct after ischemia. Lymphocytes are localized mainly at the periphery (edge) of the infarcted area and some of them invaded the core of the lesion. Lymphocytes in the acclimated rats showed less density at the edge of the ischemic lesion compared with controls. (**E–H**) 10x images of ED1-positive microglia/macrophages in the control and hypoxia acclimated groups at the core and periphery of the infarct. There was a decrease of ED1 positive cells in the stroke lesion of acclimated rats when compared with non-acclimated rats. 10x images were shown to help give the reader a sense of cell density (scale bar  = 200 µ).

In all cases macrophage and lymphocyte infiltration was present 48 h post-ischemia in the infarct regions. Qualitatively, **Fig. 3** shows a higher number of T-cells and macrophages were located in the peri-infarct region relative to the core. The density of inflammatory cells appeared higher in the control animals.

**Figure 4:**
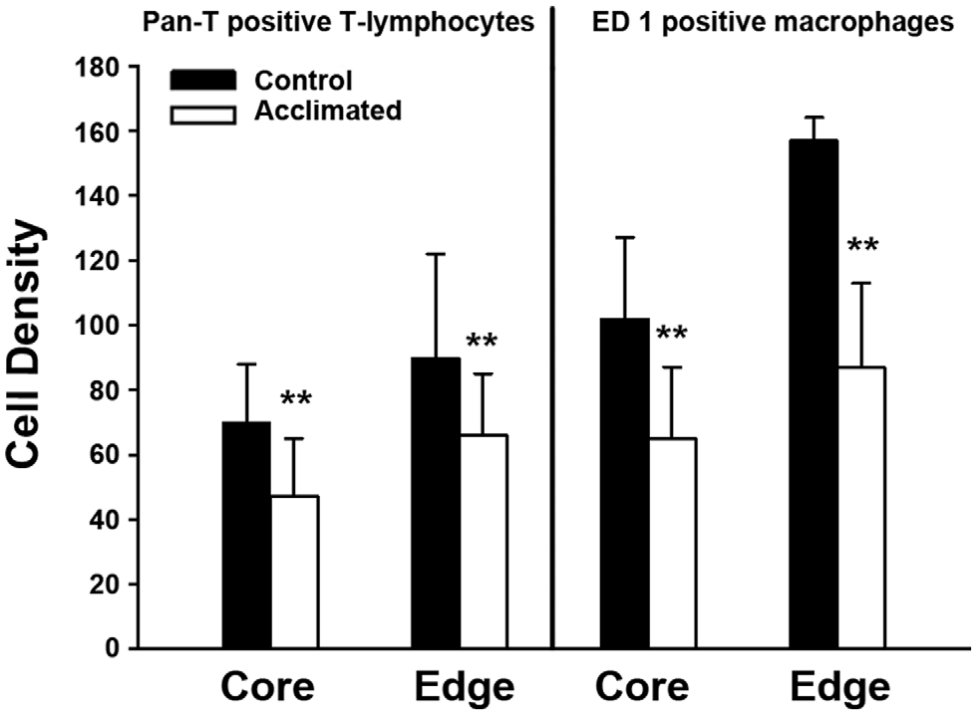
Density of Pan-T-cell lymphocytes and ED1 positive microglia/macrophages in the infarcted region of control (closed bars) and hypoxia acclimated (open bars) animals. Regions were counted in a blinded fashion in the periphery (edge) of the infarcted region as well as in the center or core. The edge and core were arbitrarily chosen by the reader. Cell Density = number/field area (0.264 mm^2^). There was a significant reduction of T-cells (*p<0.05) and ED1-positive cells (**p<0.01) in the hypoxia acclimated group compared with controls (mean±S.D.).

The figures provide a qualitative assessment, while counting of cells in the sections provided a quantitative assessment of the reduced number of infiltrating inflammatory cells. There was a 27–33% reduction in lymphocyte infiltration and a 35–45% reduction of ED1+ macrophages in the ischemic region in the hypoxia acclimated group compared to controls (**Fig. 4**).

## Discussion

This study shows that the brain can be pre-adapted with long term (weeks) exposure to chronic hypoxia in order to improve outcome from a given exposure to ischemia (stroke). This protective effect differs in timing from standard “hypoxic preconditioning” [8], [9], [12], [13], [15], [32]. Such preconditioning models use a few hours of exposure to hypoxia, or a few minutes of exposure to ischemia, and the stimulus is given within hours to a few days of the actual ischemic event [12], [13], [15], [32], [33]. In our model, after 3 weeks of exposure to hypoxia, HIF-1α and VEGF had returned to baseline [21], suggesting that the other protective growth factors would also have had returned to baseline and that the tissue had reached a new steady state. Mechanisms that protect cells after acute hypoxia preconditioning, particularly those which are evident in cell cultures [13] might not play as large a role after these factors have returned to baseline.

This time-frame is interesting as it is long enough to allow for hypoxia induced morphological changes and remodelling of the brain to make it more hypoxia tolerant. Among the hypoxia induced changes is an increased capillary density, which increases the capacity for maintaining oxygen delivery during hypoxia [2], [4]. The increased capacity, and possibly increased collateral flow, is one potential mechanism of protection. A 30% increase in capillary density in layers IV and V of the cortex was reported in this study, similar to the 57% [2], 31% [34] and 29% [35] previously reported.

Adaptations which increase capillary density and hematocrit will also increase PtO_2_ [36]. The current study showed a 30% increase in PtO_2_ with hypoxia acclimation. A previous study using electron paramagnetic resonance and undertaken near sea-level reported a 200% increase in cortical PtO_2_ [3]. A study using the similar methods as in the current paper, and also undertaken in Calgary, reported an increase of 35% [30]. The larger increases in the study undertaken at sea level could be due to the fact that control animals in the present study were living at approximately 1000 m already, and may have already undergone a level of hypoxia acclimation. This would suggest that stimulation of hypoxia responses would result in an even greater relative protective effect among lower altitude populations.

We hypothesize that increased oxygen levels and increased capillary density are key factors relating to the improved outcome from ischemia. Ventilation with high oxygen partial pressures, delivered during or after ischemia, has been shown to improve outcome from ischemia in rats [37]. Animals which hibernate are adapted to hypoxia and are also more resistant to ischemia induced brain damage [38], [39]. These animals have increased oxygen levels in brain compared to controls [40]. Exercise increases capillary density [41] and is protective against ischemic damage [41], [42]. It is of major importance to determine if increased oxygen levels improve outcomes as the data on oxygen administration in patients is not conclusive [43].

There is an additional potential mechanism of protection that relates to increased vascular density. Neural stem cells have been shown to be associated with the microvasculature. They also proliferate in a mildly hypoxic environment [44], [45]. The weeks of hypoxia could stimulate proliferation and the increase in vascular density could result in a proportional increase in the concentration of neural stem cells.

Long term intermittent hypoxic preconditioning resulted in less expression of the endothelial inflammatory markers of e-selectin reversible ischemia in rats [11]. Some macrophage and microglial response after injury is necessary for scavenging the necrotic debris facilitating the plasticity [46]. However, excess infiltration of leukocytes into the brain is detrimental and therapies that prevent the leukocyte infiltration during the acute phase after ischemia are neuroprotective [47], [48]. The reduced inflammatory response is additional evidence that the damage caused by ischemia was reduced in the acclimated group.

One obvious question is whether people living at altitude have a higher incidence of stroke or a reduced severity. There is a limited body of work on incidence, but it would appear that the number of strokes actually increases at altitude for a given population. This has been reported for people living at altitude as well as for groups moving from a lowland to highland area [49], [50]. It is likely that this increased incidence relates in part to the increased hematocrit at altitude [51]. Our data cannot be used to comment on incidence of stroke in high altitude populations. However we would predict that for a given vessel occlusion, the severity of infarction would be reduced in the group living at altitude. We could find no data relating to outcome.

Could this neurological plasticity be harnessed to improve stroke tolerance in either the general population or in high risk patient groups? It has already been shown that short term exposure to pharmacological agents which increase HIF1-α may be neuroprotective [17], [52]. A broad spectrum of transcriptional changes occurs on exposure to hypoxia, many of which could provide some level of protection against hypoxic/ischemic damage in the brain [53] if correctly stimulated. Such an intervention may be useful in high risk patients such as those who have had a transient ischemic attack.

As stroke patients are often older, it will be important to understand the effect of aging on mechanisms of protection. The increase in HIF-1α observed in the brain with hypoxia exposure is reduced in the older animal [54], [55], [56]. However, in studies on both rat and mouse brain, VEGF was significantly elevated in response to hypoxia, and the increase in capillary density was similar to that in the younger brain [54], [55]. These data indicate that the pathways regulating hypoxia response may differ in the aged brain, and the role of HIF-1α as a master switch may be reduced.

This study shows that chronic hypoxia exposure provides significant protection against hypoxic/ischemic damage. This naturally stimulated plasticity results in reduced inflammatory response, infarct volume, and behavioural dysfunction. The protection is likely to relate, in part, to an increased capillary density and tissue oxygen levels. This observation supports considering pre-activation of genes as a method of providing hypoxia tolerance and, in so doing, reducing stroke severity.
